# The effects of resistance training on postural control in children and adolescents: a systematic review and meta-analysis

**DOI:** 10.3389/fphys.2026.1928723

**Published:** 2026-10-28

**Authors:** Sichen Zhou, Cuiyue Kong, Weihao Shi, Tao Zhou, Yuxiong Xu, Yizhuo Yang, Hanan Song, Yanli Yu, Wanli Zang

**Affiliations:** 1 Sports & Medicine Integration Research Center (SMIRC), Capital University of Physical Education and Sports, Beijing, China; 2 Institute of Artificial Intelligence in Sports, Capital University of Physical Education and Sports, Beijing, China; 3 School of Physical Education, Soochow University, Suzhou, Jiangsu, China

**Keywords:** children and adolescents, dynamic balance, meta-analysis, postural control, resistance training, static balance

## Abstract

**Objective:**

To evaluate the effects of resistance training on postural control in children and adolescents.

**Methods:**

PubMed, Cochrane Library, Embase, Scopus, and Web of Science were searched from inception to April 2026 for randomized controlled trials comparing resistance training with non-resistance control conditions. Standardized mean differences (SMDs) and 95% confidence intervals (CIs) were pooled using random-effects models. Subgroup analyses for dynamic balance examined training modality, intervention duration, population characteristics, and age. Certainty of evidence was assessed using the GRADE approach.

**Results:**

Twelve trials involving 557 participants were included. Resistance training showed positive effects on static balance (SMD = 0.36, 95%CI[0.06,0.65], *p* < 0.05) and dynamic balance (SMD=0.56, 95%CI[0.23,0.89], *p* < 0.001) in children and adolescents. Subgroup analyses suggested that core and whole-body resistance training produced significant improvements in dynamic balance, whereas lower-limb resistance training did not reach statistical significance. Training modality may partly explain between-study heterogeneity. The GRADE assessment indicated that the certainty of evidence was very low for both static and dynamic balance.

**Conclusion:**

Resistance training may improve static and dynamic balance performance in children and adolescents. These findings remain preliminary because of methodological limitations, heterogeneity, and very low certainty of evidence. Further high-quality randomized controlled trials using standardized intervention protocols and outcome measures are needed to confirm the optimal training characteristics for improving postural control.

**Systematic Review Registration:**

https://www.crd.york.ac.uk/PROSPERO/view/CRD420261375407, identifier CRD420261375407.

## 1 Introduction

Childhood and adolescence represent critical periods for the rapid development of neuromuscular function, motor control, and physical activity behaviors. Postural control provides an essential foundation for standing, walking, running, jumping, changing direction, and participating in sports. Its development is influenced by multiple factors, including sensory integration, central nervous system regulation, muscle strength, joint stability, and motor experience (Horak, 2006; Chiba et al., 2013; Shanbhag et al., 2023; Schmidgen et al., 2024). Balance is an important measurable manifestation of postural control and is commonly categorized into static balance and dynamic balance. Static balance primarily reflects an individual’s ability to maintain the stability of the body’s center of mass under stable support conditions, whereas dynamic balance emphasizes the ability to regulate posture and maintain or restore stability during movement, center-of-mass displacement, changes in support conditions, or external perturbations. Because static and dynamic balance differ in sensory integration, neuromuscular control, and task demands, they show a degree of task specificity and cannot be considered fully interchangeable (Kiss et al., 2018). Examining both static and dynamic balance outcomes are important for comprehensively evaluating the potential effects of exercise interventions on postural control development in children and adolescents (Liu et al., 2024).

Resistance training (RT) is an important exercise intervention for promoting neuromuscular development in children and adolescents. Through body weight, free weights, weight machines, elastic bands, or other forms of external resistance, resistance training can induce adaptations in muscle strength, motor unit recruitment, muscle activation efficiency, muscular coordination, and joint stability (Zaricznyj et al., 1980; Faigenbaum et al., 2009; Lloyd et al., 2014). These adaptations are closely related to postural control. Greater lower-limb strength and joint stability may help individuals maintain body stability during standing, movement, and center-of-mass displacement. Core stability, multi-joint coordination, and the capacity for rapid muscle activation may also provide an important neuromuscular basis for dynamic balance tasks. Previous systematic reviews and meta-analyses in adults and older adults have suggested that resistance training may improve balance-related performance, potentially through improvements in muscle strength, neuromuscular coordination, and functional stability (Šarabon and Kozinc, 2020; Khodadad Kashi et al., 2023). However, these findings cannot be directly extrapolated to children and adolescents because postural control during childhood and adolescence is influenced by ongoing maturation, sensory integration, motor experience, and neuromuscular development (Schmid et al., 2005). Existing evidence suggests that, when appropriately designed and supervised, resistance training is feasible and safe for children and adolescents and can improve body composition (Poon et al., 2026), muscular fitness (Chaabene et al., 2025), athletic performance (Lesinski et al., 2016), and physical activity levels. Childhood and adolescence may represent an important window of opportunity for promoting the development of postural control through exercise interventions. The motor abilities and neuromuscular control foundations established during this period not only affect physical activity participation and motor skill acquisition in youth, but may also contribute to physical function and health reserves in adulthood and later life through pathways related to physical fitness development, bone mass accrual, and neuromuscular function (Telama, 2009; Faigenbaum and Myer, 2010; Mickle et al., 2011).

Despite evidence supporting the potential benefits of resistance training for balance-related outcomes, its effects on postural control in children and adolescents remain unclear. Previous systematic reviews and meta-analyses have investigated the effects of resistance training on physical performance and fitness-related outcomes in youth populations, including young athletes (Lesinski et al., 2016). Evidence specifically focusing on postural control outcomes in children and adolescents remains limited. Existing randomized controlled trials have reported variable findings (Ryan et al., 2020; Elnaggar et al., 2022), which may be attributable to differences in participant characteristics, baseline physical abilities, resistance training protocols, intervention duration, and balance assessment methods. Furthermore, static and dynamic balance represent distinct components of postural control and may respond differently to resistance training; these outcomes have not been comprehensively synthesized in previous quantitative analyses. This systematic review and meta-analysis aimed to evaluate the effects of resistance training on static and dynamic balance outcomes in children and adolescents based on randomized controlled trials (RCTs). We hypothesized that resistance training would improve balance performance in this population. In addition, we explored whether training modality, intervention duration, and age could modify the effects of resistance training on dynamic balance outcomes, thereby providing evidence to inform the development of effective resistance training strategies for improving postural control in children and adolescents.

## 2 Methods

This systematic review and meta-analysis was reported in accordance with the Preferred Reporting Items for Systematic Reviews and Meta-Analyses (PRISMA) 2020 statement (**Figure 1**). The protocol was registered in PROSPERO (registration number: CRD420261375407).

**Figure 1:**
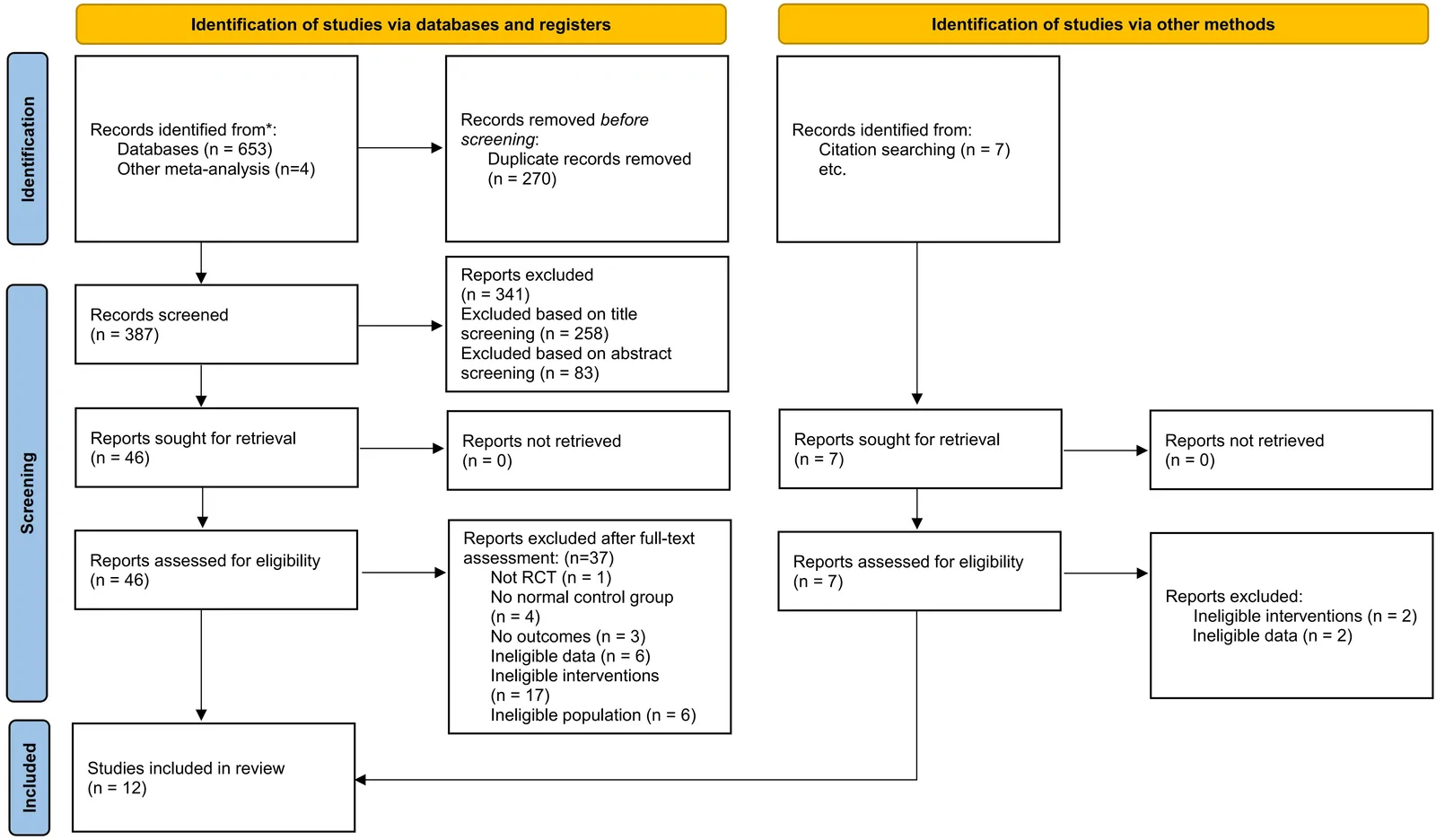
The flowchart based on PRISMA standards.

### 2.1 Search strategy

A comprehensive electronic search was conducted in PubMed, Embase, Scopus, The Cochrane Library, and Web of Science to identify randomized controlled trials relevant to the study objective. Searches were performed from database inception to April 2026. The search strategy combined controlled vocabulary and free-text terms related to the population (children, adolescents, and pediatric populations), intervention (resistance training and strength training), outcomes (postural control, postural balance, and body equilibrium), and study design (randomized controlled trials). Detailed search strategies for each database can be found in **Supplementary Tables 1.1–1.5**.

### 2.2 Inclusion criteria

This study follows the PICOS (Participants, Intervention, Comparison, Outcome, Study Design) framework, and the inclusion criteria are as follows:

1. Participants: Children and adolescents, including preschool children, school-aged children, and adolescents, as well as clinical populations within this age category, were eligible for inclusion. No specific minimum or maximum age cutoff was applied during study selection; eligibility was determined according to the population characteristics reported in each study. No restrictions were imposed regarding sex or ethnicity.
2. Interventions: The operational definition of resistance training was developed based on the principles outlined in the International Consensus on Youth Resistance Training (Lloyd et al., 2014). Resistance training was defined as a structured exercise program involving repeated voluntary muscle contractions performed against an external load or body weight, with the primary purpose of improving muscular strength, power, neuromuscular function, or related physical performance outcomes. Studies involving only balance training, flexibility training, aerobic exercise, or combined interventions in which the independent effects of resistance training could not be isolated were excluded. For studies with multiple intervention arms, only the resistance training arm was included. When a single study reported multiple independent resistance training groups, these groups were treated as separate comparisons.
3. Comparison: The control group received standard physical rehabilitation, regular physical education classes, physical therapy, or other non-resistance training interventions.
4. Outcomes: This review focused specifically on static and dynamic balance as measurable outcomes reflecting key aspects of postural control rather than encompassing all dimensions of postural control. Eligible studies were required to assess static or dynamic balance using validated balance assessment tools or established measurement protocols, and the corresponding outcome scores were extracted for quantitative synthesis.
5. Study design: Randomized controlled trials (RCTs).

### 2.3 Exclusion criteria

1. Studies were excluded if the study population did not include children and adolescents or did not meet the predefined PICOS criteria.
2. Intervention: resistance training was not the only intervention component, for example, resistance training combined with balance training or other exercise modalities.
3. Studies where the full text or original data could not be obtained.
4. Duplicate publications or studies with unclear data descriptions.
5. Studies that did not assess the primary outcomes of interest.
6. Studies were excluded when means and standard deviations were unavailable and could not be derived from the reported data or obtained from the corresponding author.
7. Non-peer-reviewed literature, including English articles, abstracts, reviews, and conference papers.

### 2.4 Study selection and data extraction

Two reviewers (SC-Z and CY-K) independently conducted the literature screening and data extraction. Any disagreements were resolved through discussion with a third reviewer (Shi). All retrieved records were imported into EndNote for duplicate removal. After deduplication, studies were screened according to the predefined inclusion and exclusion criteria.

Two reviewers (SC-Z and YX-X) entered the extracted study information and data into Microsoft Excel and cross-checked the entries for accuracy. The extracted information included the first author, year of publication, country of the study, study design, basic participant characteristics, including sex, age, and health status or clinical condition, intervention characteristics, including sample size, assessment scale or measurement method, type of resistance training, intervention duration, exercise intensity, and training frequency, as well as effect measures and data types. Studies including participants across multiple developmental stages were retained when the overall reported age range was within the predefined eligibility criteria. The potential influence of developmental differences was considered during interpretation and discussed as a limitation. For studies with a small proportion of participants outside this range, eligibility was determined based on the available age information and reported characteristics of the analyzed sample.

### 2.5 Risk of bias and quality assessment

The risk of bias of the included studies was independently assessed by two reviewers(SC-Z and CY-K) using the Cochrane Risk of Bias 2 (RoB 2) tool, which evaluates five domains: bias arising from the randomization process, bias due to deviations from intended interventions, bias due to missing outcome data, bias in measurement of the outcome, and bias in selection of the reported result (Sterne et al., 2019). Detailed judgments and supporting rationales for each RoB 2 domain and each included study are provided in **Supplementary Table 3**. The Physiotherapy Evidence Database (PEDro) scale was additionally applied as a supplementary measure to characterize the methodological quality and reporting features of the included randomized controlled trials (Moseley et al., 2019). The PEDro scale consists of 11 items assessing eligibility criteria, random allocation, concealed allocation, baseline comparability, blinding, outcome assessment, follow-up, intention-to-treat analysis, between-group comparisons, and measures of variability. The first item, which assesses external validity based on eligibility criteria, is not included in the total score; therefore, the maximum possible PEDro score is 10 points. PEDro scores were interpreted as follows: scores of 4-5 indicated low methodological quality, 6-8 indicated moderate methodological quality, and 9-10 indicated high methodological quality (Ramachandran et al., 2021). The PEDro scale was used only to describe the methodological characteristics and reporting quality of the included studies and was not considered a criterion for study eligibility, exclusion, or quantitative synthesis. Any disagreements between reviewers were resolved through discussion and consensus with a third reviewer(YL-Y). The results of the PEDro assessment are presented in **Supplementary Table 4**.

### 2.6 Sensitivity analysis

Sensitivity analyses were conducted to evaluate the robustness of the pooled estimates and to explore whether methodological or clinical heterogeneity influenced the results. First, a leave-one-out analysis was performed by sequentially removing each study from the meta-analysis to assess the influence of individual studies on the pooled effect estimates. Second, sensitivity analyses were conducted by excluding studies with lower methodological quality (PEDro score <6), studies involving specific population characteristics (clinical populations and preschool-aged children), and studies using different comparator conditions (physical education or physical therapy). The stability of the pooled effect estimates was assessed by comparing the results of these sensitivity analyses with those of the primary analyses.

### 2.7 Certainty of evidence assessment

Two authors (SC-Z and WH-S) independently assessed the certainty of evidence for each meta-analysis using the Grading of Recommendations Assessment, Development and Evaluation (GRADE) approach (Balshem et al., 2011). The certainty of evidence was downgraded when concerns were identified in the following domains: (1) risk of bias, defined as more than 75% of the included studies not being classified as having a low risk of bias; (2) inconsistency, indicated by an I² statistic greater than 50%; (3) imprecision, judged according to the total sample size, number of included studies, width of the 95% confidence interval, and whether the confidence interval crossed the line of no effect or included effects in clinically different directions; (4) indirectness, assessed according to whether the study populations, intervention types, comparator conditions, and outcome measures were fully consistent with the research question; and (5) publication bias was assessed by considering the comprehensiveness of the literature search, potential publication restrictions, and small-study effects. Funnel plots and statistical tests were considered exploratory analyses when sufficient studies were available. A Summary of Findings (SoF) table was generated to present the main outcomes, including the number of included studies and participants, pooled effect estimates, 95% confidence intervals, and the overall certainty of evidence according to the GRADE framework.

### 2.8 Subgroup analyses

Subgroup analyses were conducted for outcome measures exhibiting significant heterogeneity (I²≥50%) to investigate the potential sources of variation. Training programs are categorized as core resistance training, lower-body resistance training or whole-body resistance training, based on the primary anatomical and functional focus of each intervention (Stricker et al., 2020). In accordance with pediatric resistance training guidelines, which specify 8 weeks as a clinical reference point (Lloyd et al., 2014), the duration of intervention was categorized into three groups: <8 weeks, 8 weeks and >8 weeks. Because the included studies covered a broad developmental period from preschool childhood to adolescence, exploratory age subgroup analyses were conducted using a mean age threshold of 10 years. This cutoff was selected based on the WHO definition of adolescence beginning at 10 years and was considered an exploratory approach to examine potential developmental differences rather than a strict classification of developmental stages (G.A.f.M.o.A.H.A.G. (GAMA) and World Health Organization, 2024). Based on clinical relevance, the study participants were categorized into children and adolescents with clinical conditions or special needs, and healthy children and adolescents (including adolescent athletes).

### 2.9 Statistical analysis

Meta-analyses were performed using Stata version 14 and Review Manager version 5.4. Statistical heterogeneity was assessed using Cochran’s Q test and quantified using the I² statistic, with values of approximately 25%, 50%, and 75% indicating low, moderate, and high heterogeneity, respectively (Higgins et al., 2003). Given the expected clinical and methodological heterogeneity among studies, random-effects models were used for all pooled analyses. All outcomes were continuous variables, and standardized mean differences (SMDs) with 95% confidence intervals (CIs) were calculated because different assessment instruments were used across studies. SMD values of 0.2, 0.5, and 0.8 were interpreted as small, moderate, and large effects, respectively. When multiple balance outcomes were reported within the same outcome category, effect estimates were combined to avoid unit-of-analysis errors. Publication bias was explored using funnel plots, Egger’s regression test, and Begg’s rank correlation test when the number of included studies was sufficient. These analyses were considered exploratory because their statistical power is limited when few studies are available.

## 3 Results

### 3.1 Search results

A total of 653 records were identified through database searching, including PubMed (n=30), Scopus (n=166), Embase (n=398), The Cochrane Library (n=25), and Web of Science (n=34). Four additional records were identified from other relevant sources, including previous meta-analyses. After removal of 270 duplicate records, 387 records remained for title and abstract screening. During the title and abstract screening process, 341 records were excluded, including 258 records excluded based on title screening and 83 records excluded based on abstract screening (**Supplementary Table 2**). The remaining 46 reports were retrieved for full-text assessment of eligibility. After full-text assessment, 37 reports were excluded for the following reasons: not being randomized controlled trials (n=1), no normal control group (n=4), no relevant outcomes (n=3), ineligible data (n=6), ineligible interventions (n=17), and ineligible population (n=6). In addition, seven reports were identified through citation searching, all of which were retrieved and assessed for eligibility. Four reports were excluded due to ineligible interventions (n=2) and ineligible data (n=2). Finally, 12 studies were included in the systematic review and meta-analysis (**Figure 1**).

### 3.2 Characteristics of included studies

This meta-analysis included 12 studies published between 2011 and 2025 (**Supplementary Table 5**), with a total of 557 participants (Granacher et al., 2011; Aly and Abonour, 2016; Kordi et al., 2016; Ozmen and Aydogmus, 2016; El-Shamy, 2017; Larsen et al., 2018; Alsakhawi and Elshafey, 2019; Hammami et al., 2019; Elnaggar et al., 2021; Elnaggar et al., 2022; Park et al., 2024; Tuncer et al., 2025). The studies were conducted across nine countries, including Egypt (n=2), Saudi Arabia (n=2), Turkey (n=2), the United Kingdom (n=1), Germany (n=1), Canada (n=1), South Korea (n=1), Tunisia (n=1), and Iran (n=1). All included interventions were resistance training programs. The mean intervention duration was 11.08 ± 9.30 weeks, and the mean training frequency was 2.75 ± 0.87 sessions per week. The included studies involved participants ranging from 4 to 18 years of age, covering preschool children, school-aged children, and adolescents. This age range reflects the characteristics of the included studies rather than a predefined eligibility criterion.

### 3.3 Risk of bias assessment of the included studies

All 12 included studies reported random allocation, and nine studies provided details of the randomization procedures (Granacher et al., 2011; Aly and Abonour, 2016; Kordi et al., 2016; El-Shamy, 2017; Larsen et al., 2018; Elnaggar et al., 2021; Elnaggar et al., 2022; Park et al., 2024; Tuncer et al., 2025), primarily using computer-generated random sequences or sealed-envelope methods. Two studies explicitly reported a single-blind design (Kordi et al., 2016; Elnaggar et al., 2022). The results of the RoB 2 assessment are presented in **Figure 2**. Overall, several included studies were judged as having some concerns or a high risk of bias across specific domains. Most studies showed low risk of bias in the domains of missing outcome data and selection of the reported result. Detailed domain-level judgments and supporting rationales for each study are provided in **Supplementary Table 3**. Methodological quality was additionally assessed using the PEDro scale (**Supplementary Table 4**). The PEDro scores ranged from 5 to 9, with the majority of studies demonstrating moderate methodological quality. One study was classified as high quality (Park et al., 2024), ten as moderate quality (Granacher et al., 2011; Aly and Abonour, 2016; Kordi et al., 2016; Ozmen and Aydogmus, 2016; El-Shamy, 2017; Larsen et al., 2018; Alsakhawi and Elshafey, 2019; Elnaggar et al., 2021; Elnaggar et al., 2022; Tuncer et al., 2025), and one as low quality (Hammami et al., 2019).

**Figure 2:**
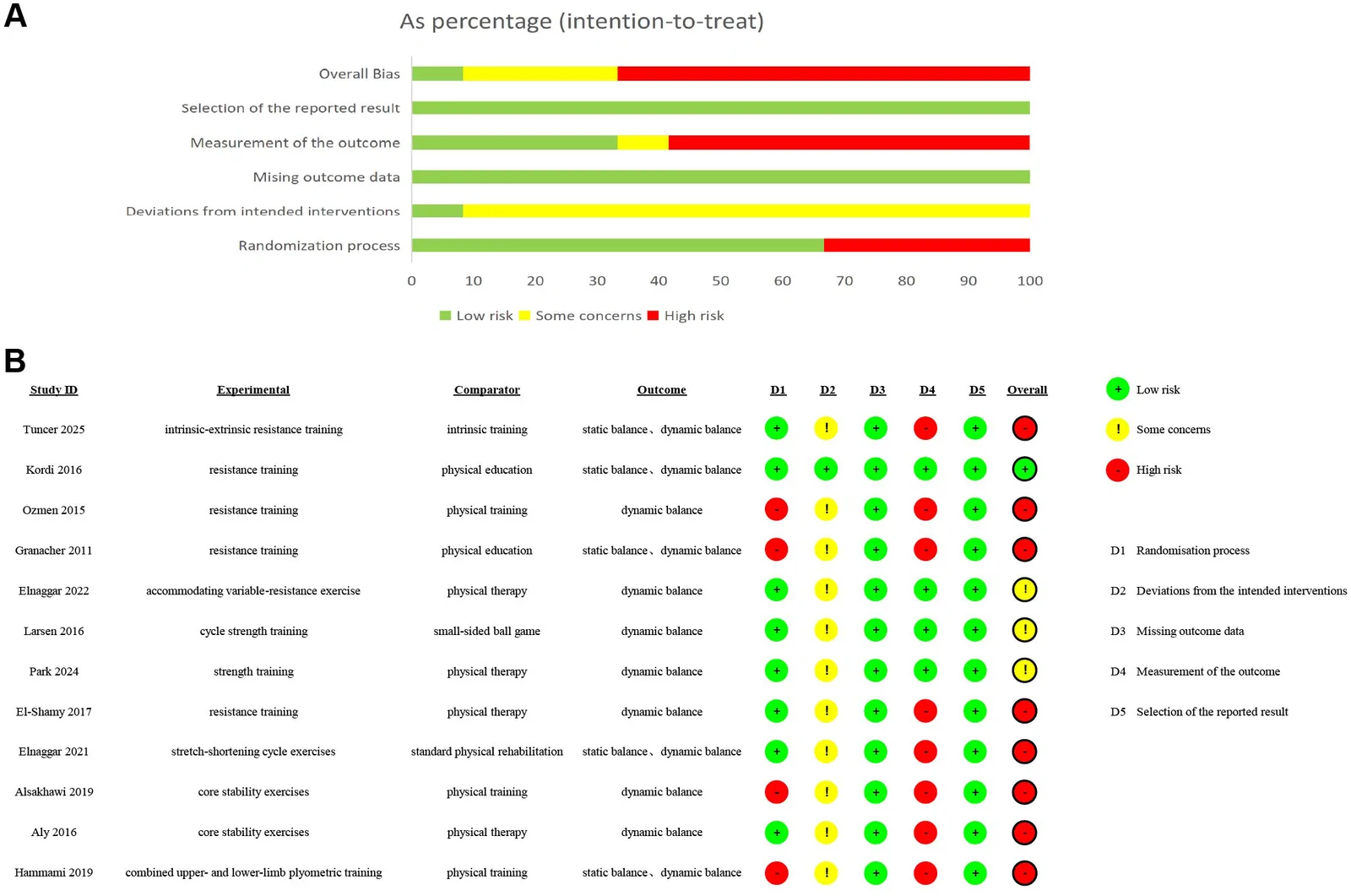
Risk of bias assessment.

A:Summary of bias risk assessment. B: The data on the risk of bias assessment for each study.

### 3.4 Publication bias

Publication bias was explored using funnel plot inspection and asymmetry tests as exploratory analyses (**Figure 3**). For static balance, only five studies were included; therefore, funnel plot asymmetry tests were considered exploratory and interpreted cautiously because of the limited statistical power to detect small-study effects. For dynamic balance, funnel plot asymmetry tests did not provide clear evidence of small-study effects; however, these findings should also be interpreted cautiously because statistical tests have limited ability to reliably exclude publication bias when the number of studies is limited. Considering the comprehensive search strategy, inclusion of multiple databases, and the absence of restrictions based on publication status or publication type, publication bias was not downgraded in the GRADE assessment. The possibility of publication bias cannot be completely excluded.

**Figure 3:**
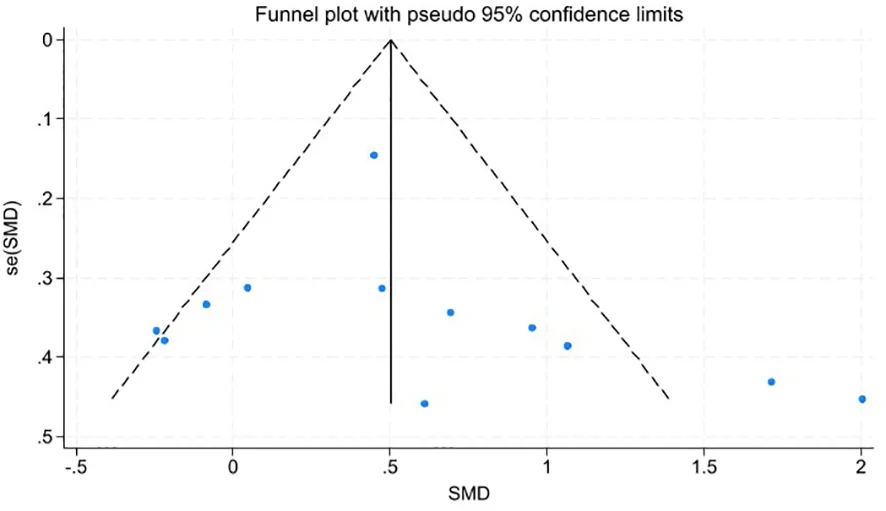
Funnel plot of resistance training on dynamic balance (exploratory analysis).

### 3.5 Sensitivity analyses

Leave-one-out analysis showed that sequential removal of individual studies did not substantially change the pooled effect estimate, indicating that no single study had a disproportionate influence on the overall findings(**Supplementary Figure 1**).

Sensitivity analysis excluding studies with lower methodological quality (PEDro score <6) produced effect estimates comparable to the primary analysis, suggesting that the pooled results were not substantially affected by studies with lower methodological quality (**Supplementary Figure 2**).

Sensitivity analyses based on population characteristics showed that the pooled effect remained significant when only studies involving clinical populations were retained (SMD = 0.89, 95%CI[0.28,1.50]), whereas the effect was attenuated and no longer statistically significant after excluding clinical populations (SMD=0.30, 95%CI[-0.01,0.61]). These results indicate that population characteristics may contribute to heterogeneity among studies (**Supplementary Figure 3**).

Sensitivity analyses according to comparator conditions showed that studies using conventional physical therapy as the comparator yielded a significant pooled effect with lower heterogeneity (7 studies; SMD = 0.90, 95%CI[0.52,1.27]; I²=29%). Studies using usual training or physical education classes as comparators showed a smaller and non-significant effect estimate (5 studies; SMD = 0.31, 95%CI[-0.13,0.75]). These findings suggest that comparator conditions may represent another potential source of heterogeneity (**Supplementary Figure 4**).

Sensitivity analysis was conducted by excluding the two studies involving preschool-aged children from the dynamic-balance analysis. After removal of these studies, the pooled effect remained statistically significant (SMD = 0.51, 95%CI[0.20,0.82]), with moderate heterogeneity (I²=57%). The results were consistent with the primary analysis, indicating that the inclusion of preschool-aged participants did not substantially influence the overall findings (**Supplementary Figure 5**).

### 3.6 Certainty of evidence

The certainty of evidence for the primary outcomes was assessed using the GRADE approach. The Summary of Findings table (**Table 1**) presents the pooled effect estimates, number of included studies and participants, and the certainty of evidence for static and dynamic balance outcomes. According to the GRADE criteria, the certainty of evidence was very low for both static and dynamic balance outcomes (**Supplementary Table 6**). The certainty of evidence for static balance was downgraded due to concerns regarding risk of bias, indirectness, and imprecision, including methodological limitations of the included studies, differences in populations or interventions, and limited sample sizes with imprecise estimates. The certainty of evidence for dynamic balance was downgraded due to concerns regarding risk of bias, inconsistency, indirectness, and imprecision, reflecting methodological limitations, heterogeneity among studies, indirectness of evidence, and imprecise effect estimates. Publication bias was not downgraded for either outcome because the search strategy was comprehensive and no restrictions were applied based on publication status or publication type. The possibility of publication bias cannot be completely excluded due to the limited number of included studies.

**Table 1:** Summary of findings for the effects of resistance training on balance outcomes.

Outcome	No. of studies	Participants	Effect estimate	Certainty of evidence
Static balance	5 RCTs	177	SMD 0.36, 95% CI[0.06,0.65]	Very low
Dynamic balance	12 RCTs	557	SMD 0.56, 95% CI[0.23, 0.89]	Very low

CI, confidence interval; RCT, randomized controlled trial; SMD, standardized mean difference. The certainty of evidence was assessed using the Grading of Recommendations Assessment, Development and Evaluation (GRADE) approach.

### 3.7 Meta-analysis

#### 3.7.1 Effect of resistance training on static balance in children and adolescents

Five studies were included in the analysis of static balance outcomes (Granacher et al., 2011; Kordi et al., 2016; Hammami et al., 2019; Elnaggar et al., 2021; Tuncer et al., 2025). The heterogeneity test showed no significant between-study inconsistency (Q = 2.54, df=4, p=0.64, I²=0%). Given the potential clinical and methodological differences across the included studies in participant characteristics, training protocols, and outcome measurement tools, a random-effects model was still used for the pooled analysis. The results showed that, compared with control conditions, resistance training may improve static balance performance in children and adolescents, with a statistically significant difference between groups (SMD = 0.36, 95%CI[0.06, 0.65], p=0.02, I² = 0%; **Figure 4**).

**Figure 4:**
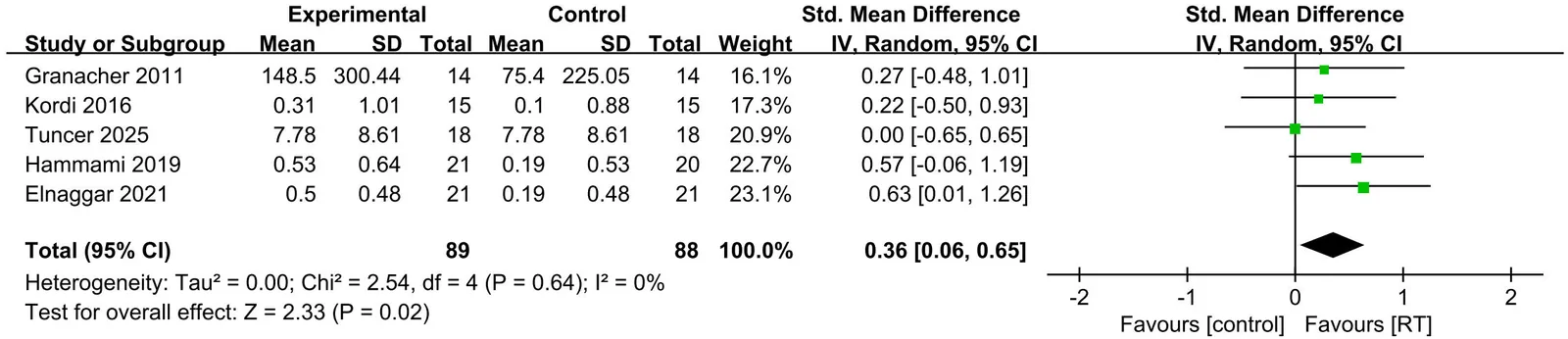
Forest plot of the effects of resistance training on static balance in children and adolescents.

#### 3.7.2 Effects of resistance training on dynamic balance in children and adolescents

Twelve studies were included in the analysis of dynamic balance outcomes (Granacher et al., 2011; Aly and Abonour, 2016; Kordi et al., 2016; Ozmen and Aydogmus, 2016; El-Shamy, 2017; Larsen et al., 2018; Alsakhawi and Elshafey, 2019; Hammami et al., 2019; Elnaggar et al., 2021; Elnaggar et al., 2022; Park et al., 2024; Tuncer et al., 2025). The heterogeneity test indicated substantial between-study heterogeneity (Q=34.02, df=11, p<0.001, I²=68%), suggesting the need for further exploration of potential sources of heterogeneity. A random-effects model was used for the pooled analysis. Compared with control conditions, resistance training may significantly improve dynamic balance in children and adolescents, with a statistically significant between-group difference (SMD=0.56, 95%CI[0.23, 0.89], p<0.001 I^2^=68%; **Figure 5**).

**Figure 5:**
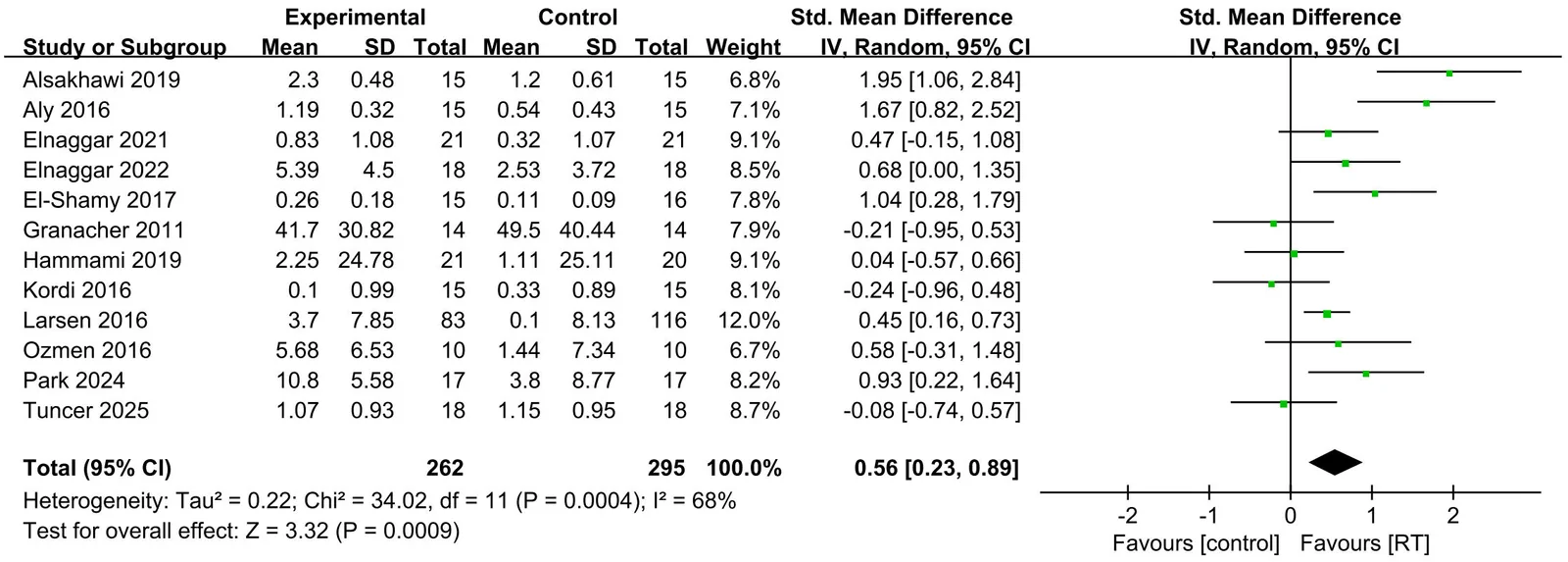
Forest plot of the effects of resistance training on dynamic balance in children and adolescents.

### 3.8 Subgroup analysis

The effects of resistance training on dynamic balance varied across intervention type. Core resistance training demonstrated a large and statistically significant pooled effect (SMD = 1.41, 95%CI[0.60,2.21], p<0.001). Whole-body resistance training also showed a significant positive effect, with the lowest within-subgroup heterogeneity (SMD = 0.41, 95%CI[0.17,0.66], p=0.001). Lower-limb resistance training showed a smaller and non-significant effect (SMD = 0.31, 95%CI[-0.13,0.76], p=0.17). The effect estimates differed marginally across training modalities (Qb=5.90 p=0.05) (**Figure 6**).

**Figure 6:**
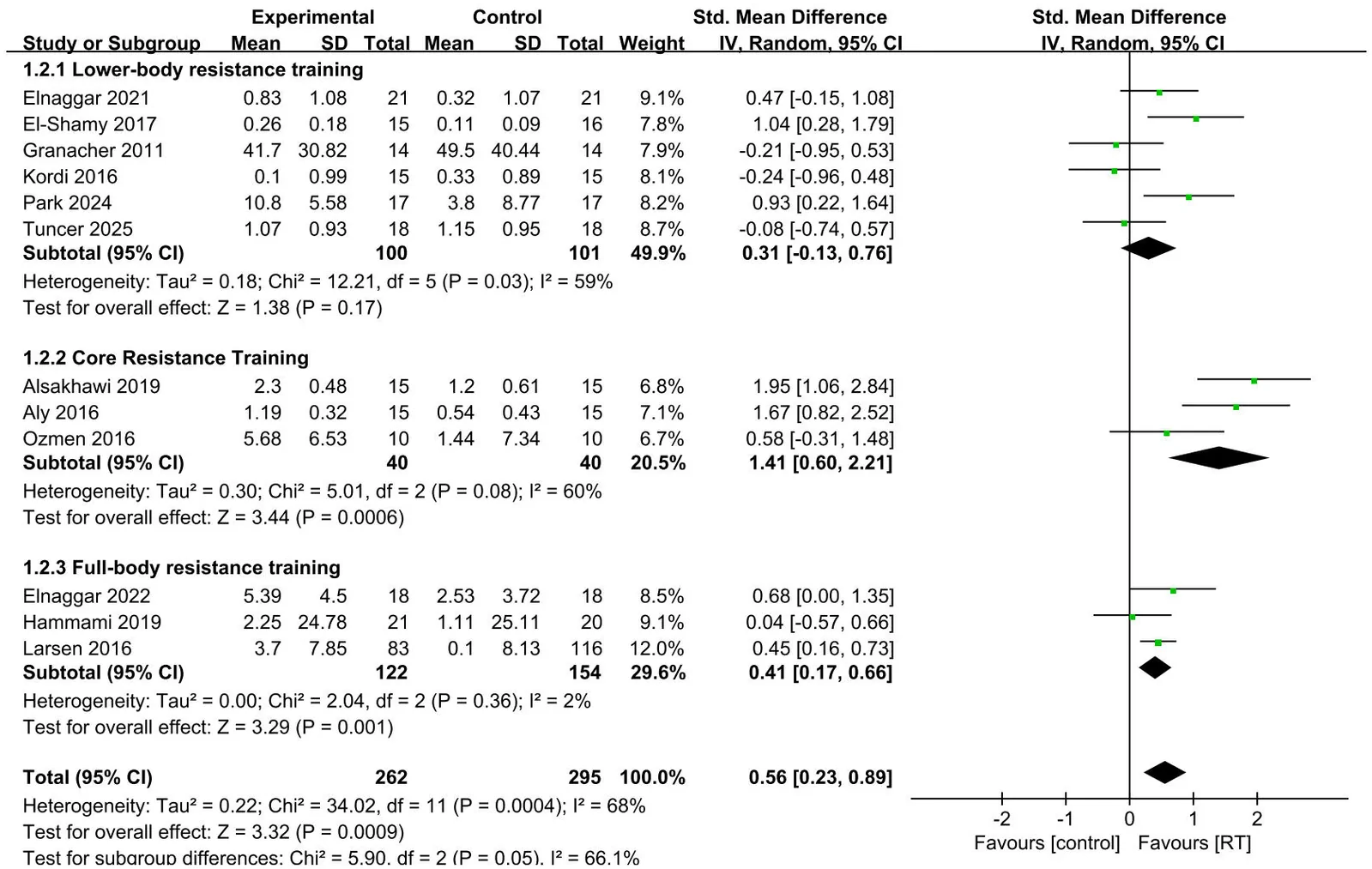
Forest plot of subgroup analysis by resistance training type.

The effects of resistance training on dynamic balance varied across intervention durations. Both shorter-duration training (<8 weeks) and 8-week training demonstrated statistically significant effects, with relatively larger pooled estimates in the shorter-duration subgroup (<8 weeks: SMD = 0.80, 95%CI[0.24,1.36], p=0.005; 8 weeks: SMD = 0.71, 95%CI[0.06,1.35], p=0.03). In contrast, interventions lasting >8 weeks showed a smaller pooled effect estimate that did not reach statistical significance (SMD = 0.33, 95%CI[-0.11,0.76], p=0.14). The test for between-subgroup differences showed no statistically significant differences across intervention durations (Qb=2.00, p=0.37) (**Figure 7**).

**Figure 7:**
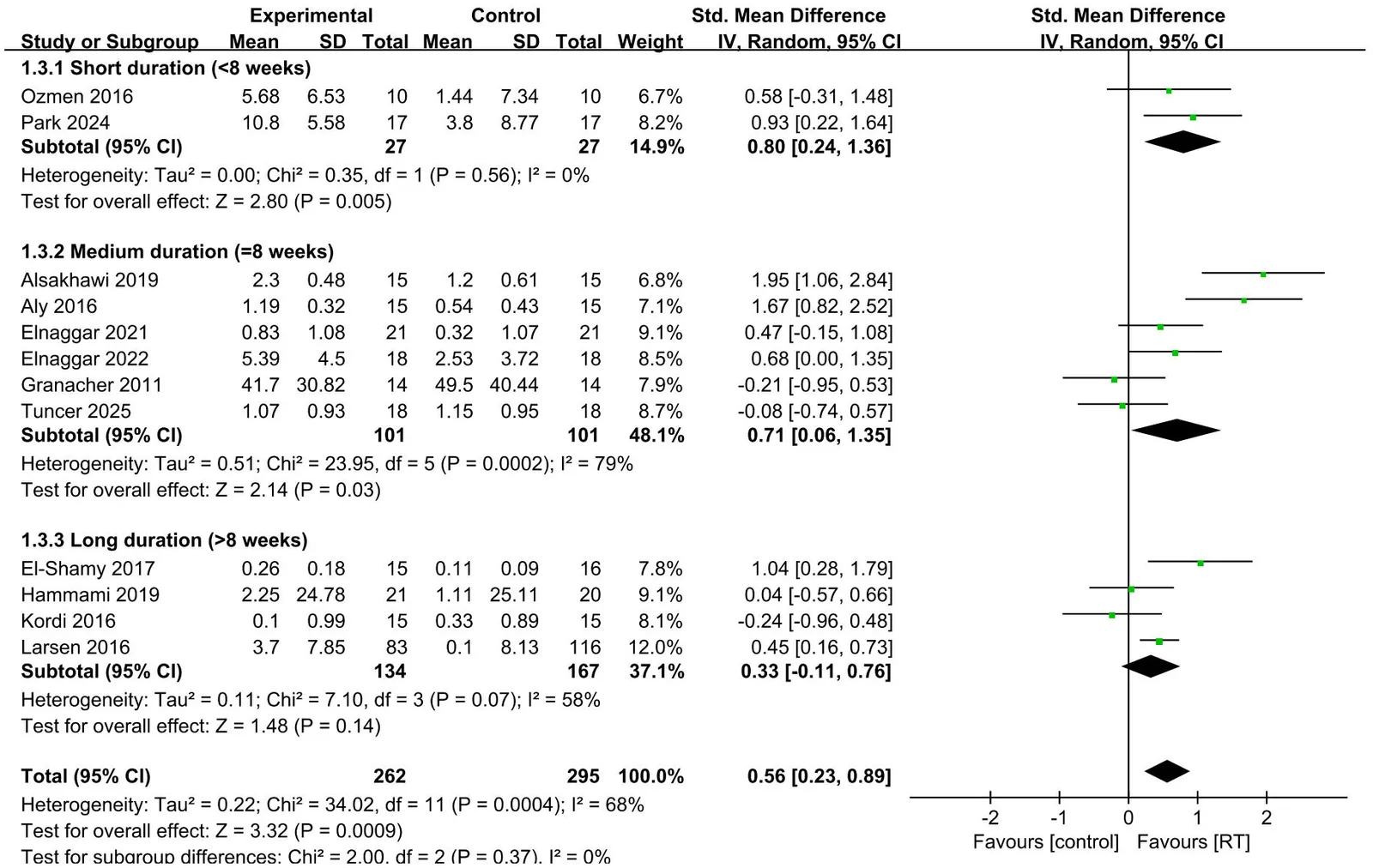
Forest plot of subgroup analysis by resistance training duration.

The effects of resistance training on dynamic balance were generally comparable between age groups. In studies with a mean participant age of <10 years, the pooled effect estimate was moderate but did not reach statistical significance, with the confidence interval crossing the line of no effect (SMD=0.57, 95%CI[-0.01,1.15], p=0.05), which showed substantial heterogeneity (I²=81%).In studies with a mean participant age of ≥10 years, resistance training was associated with a statistically significant moderate effect on dynamic balance (SMD=0.58, 95%CI[0.23,0.93], p=0.001), with relatively low heterogeneity (I²=27%). The test for between-subgroup differences showed no significant difference between the two age groups (Qb=0.00, p=0.98) (**Figure 8**).

**Figure 8:**
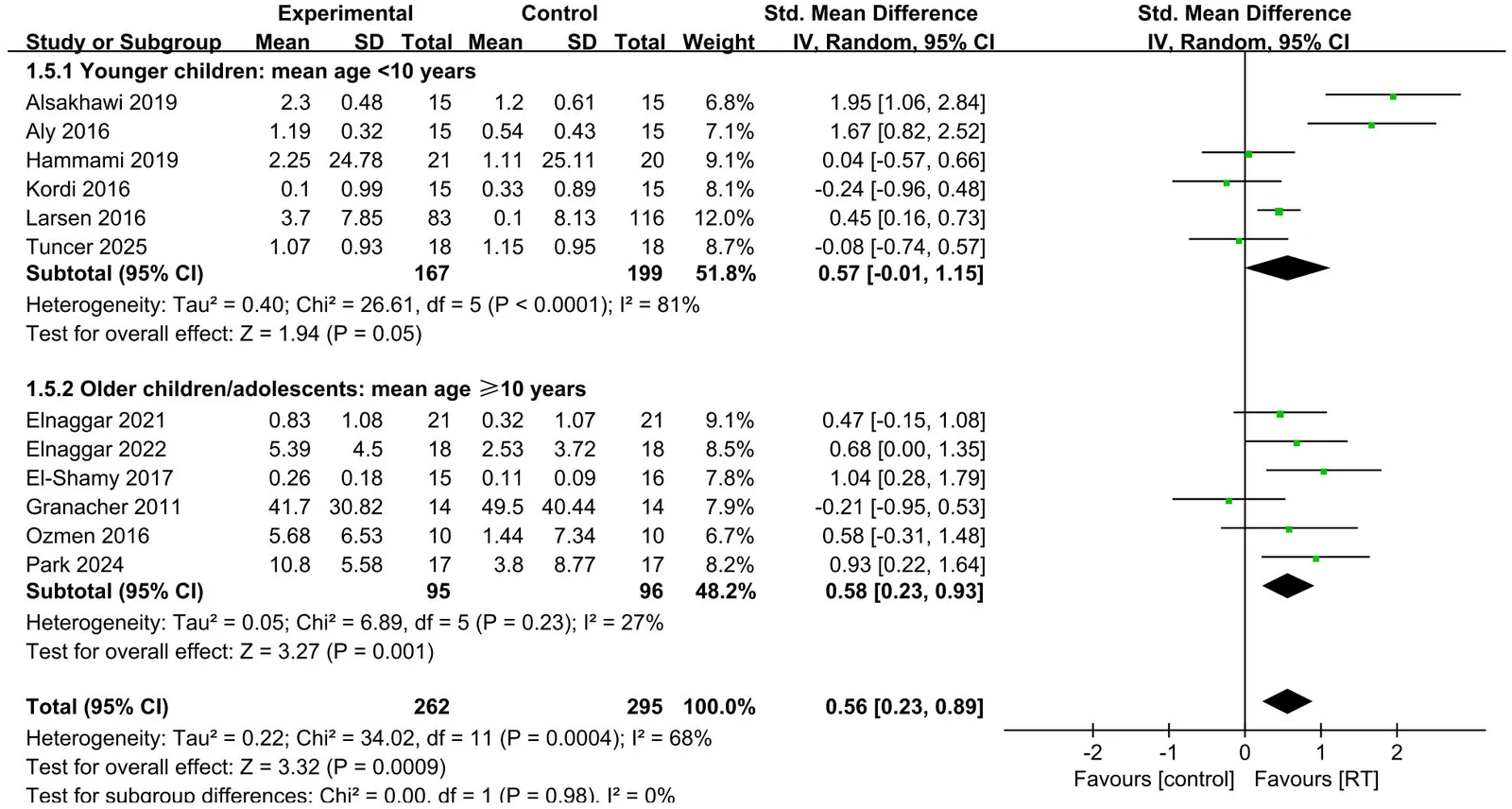
Forest plot of the age subgroup analysis of resistance training.

The effect of resistance training on dynamic balance did not significantly differ between population subgroups. In children with special needs, resistance training showed a relatively large positive effect, with a statistically significant pooled effect (SMD = 0.89, 95%CI[0.28,1.50], p=0.004). Within-subgroup heterogeneity was high (I²=75%). In healthy children, the pooled effect size was smaller and only approached statistical significance, with the confidence interval crossing the line of no effect (SMD = 0.30, 95%CI[-0.01,0.61], p=0.06). Within-subgroup heterogeneity was relatively low (I²=38%). The test for between-subgroup differences showed no statistically significant difference between children with special needs and healthy children (Qb=2.92, p=0.09) (**Figure 9**).

**Figure 9:**
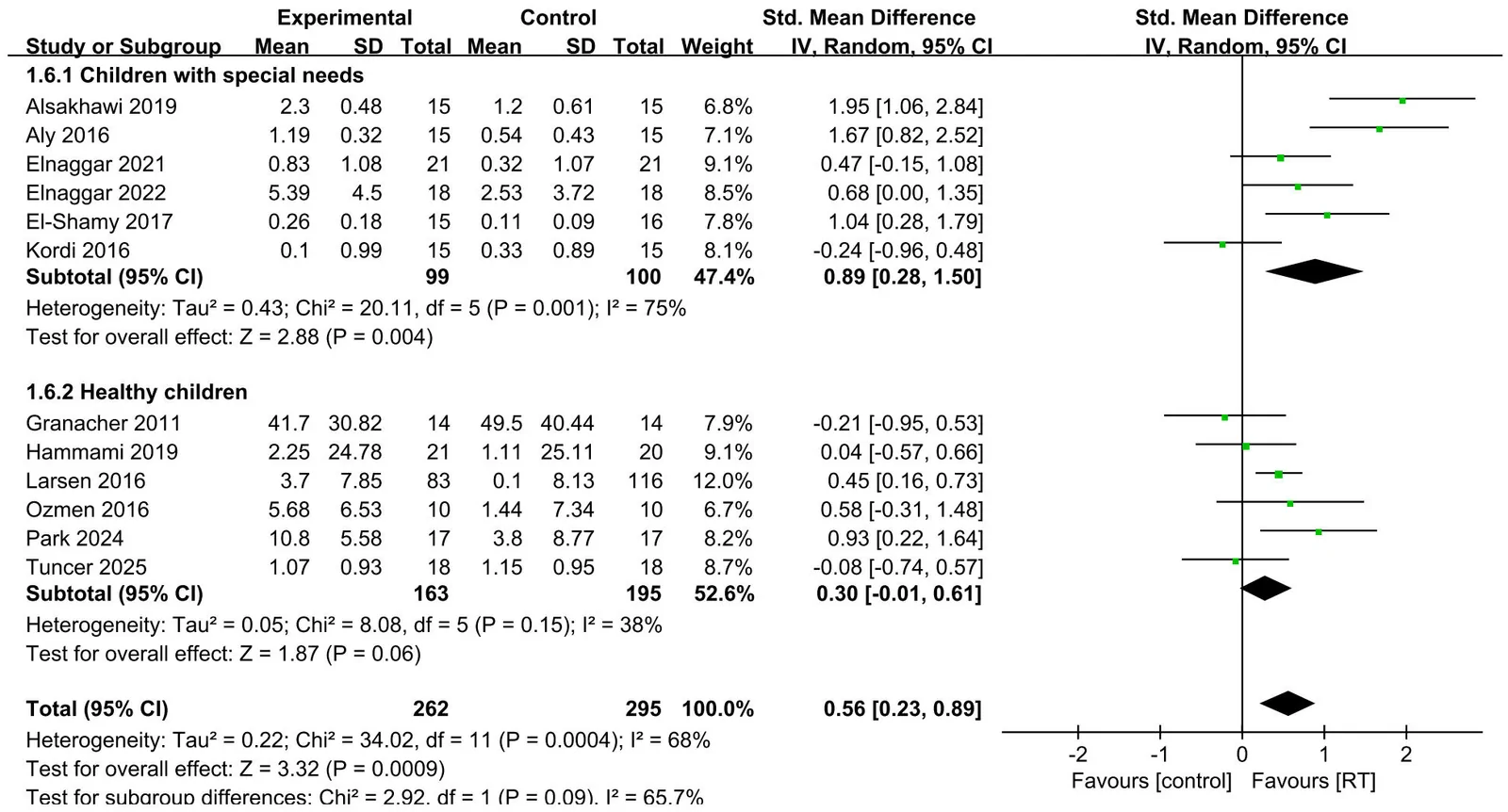
Forest plot of subgroup analysis of population characteristics.

## 4 Discussion

This meta-analysis included 12 randomized controlled trials involving 557 children and adolescents, aiming to evaluate the effects of resistance training on postural control-related outcomes. The findings suggest that resistance training may improve both static and dynamic balance performance in children and adolescents. The analysis of static balance demonstrated a favorable effect, with a relatively low level of between-study heterogeneity (SMD = 0.36, 95%CI[0.06,0.65], I²=0%). Dynamic balance also showed a positive pooled effect; however, substantial heterogeneity was observed across studies (SMD=0.56, 95%CI[0.23, 0.89], I²=69%). The potential influence of moderating factors on dynamic balance outcomes was explored in an exploratory manner and should be interpreted with caution.

Previous studies have demonstrated that resistance training exerts beneficial effects on balance performance (Šarabon and Kozinc, 2020; Lei et al., 2025; Leale et al., 2026), and the pooled findings of the present meta-analysis further support this evidence. The underlying mechanisms may be attributed to resistance training-induced improvements in muscle strength and neuromuscular adaptations (Granacher et al., 2013; Kidgell et al., 2017; Casolo et al., 2020). These adaptations may enhance static and dynamic balance performance by facilitating sensorimotor integration, optimizing neuromuscular control, and improving motor output capacity. Static balance primarily depends on the ability to maintain the body’s center of mass within a stable support base under relatively stationary conditions, requiring sustained low-level muscle activation and precise perception of body position. Resistance training may enhance static balance by increasing muscle strength and local muscular endurance, thereby expanding the force reserve required for maintaining upright posture and enabling the body to effectively counteract minor postural perturbations during standing. Furthermore, long-term resistance training may further enhance static postural control by improving proprioceptive function (Barrio et al., 2022). The muscle contractions and joint loading stimuli induced by resistance training may increase the ability to perceive changes in joint position, muscle tension, and body center of mass, thereby facilitating the integration of peripheral sensory inputs with central motor outputs. These adaptations may enhance sensory feedback from muscle spindles, Golgi tendon organs, and joint mechanoreceptors, providing additional support for postural control (Tumkur Anil Kumar et al., 2021). In contrast, dynamic balance primarily involves the active regulation of the body’s center of mass during movement, requiring the coordinated integration of visual, vestibular, and proprioceptive inputs, as well as rapid coordination of the multi-joint kinetic chain involving the ankle, knee, and hip joints (Taube et al., 2008; Welch and Ting, 2014). Resistance training may enhance dynamic stability by improving force production capacity, optimizing inter-joint coordination, and enhancing postural response ability, thereby facilitating the maintenance of stability during weight shifting and dynamic postural adjustments.

Previous evidence regarding the effects of resistance training on balance performance has primarily focused on adults and older populations, whereas systematic investigations in children and adolescents undergoing growth and development remain relatively limited (Šarabon and Kozinc, 2020). The findings of the present meta-analysis indicate that resistance training may improve postural control in children and adolescents, providing new evidence for understanding the adaptive characteristics of exercise training during developmental stages. Childhood and adolescence represent critical periods characterized by rapid development of the nervous system, sensorimotor systems, and motor control capacity. During these developmental stages, individuals may exhibit greater neuroplasticity and increased sensitivity to training stimuli that promote neuromuscular coordination, motor control, and postural regulation (Behm et al., 2008). The neuromuscular adaptations induced by resistance training may therefore be more readily translated into improvements in postural control. The exploratory findings suggest that resistance training programs incorporating multi-joint compound exercises, with an emphasis on core stability and whole-body coordination, may demonstrate a greater magnitude of effect, which is more consistent with the functional demands of postural control involving multi-joint coordination and dynamic regulation of the body’s center of mass. However, given the limited number of studies included in each training modality subgroup and the substantial heterogeneity observed within subgroups, it remains premature to determine whether any specific training approach offers a definitive advantage.

In the present meta-analysis, resistance training interventions lasting ≤8 weeks were associated with relatively greater improvements in postural control, which differs to some extent from previous studies emphasizing the potential advantages of longer-term training programs (Behringer et al., 2010). Further analyses indicated that studies involving training durations >8 weeks predominantly included children younger than 10 years of age, among whom the estimated training effects appeared relatively limited. These findings suggest a potential association between training duration and age-related factors. However, because the subgroup differences did not reach statistical significance, the presence of an interaction between age and training duration cannot be confirmed. Therefore, these findings should be interpreted as exploratory, suggesting that developmental stage may influence adaptive responses to resistance training and warrant further investigation in future studies. A preliminary interpretation of this finding can be proposed from a physiological perspective. Early improvements in postural control following resistance training may primarily result from neural adaptations, including enhanced motor unit recruitment, improved intermuscular coordination, and optimization of postural control strategies (Faigenbaum and Myer, 2010). As these neural adaptations typically occur before structural changes in muscle tissue, measurable improvements in postural control may be observed even within relatively short training periods. On the other hand, a longer training duration does not necessarily indicate a sustained increase in effective training exposure. Younger children participating in structured resistance training programs may be influenced by factors such as motivation maintenance, parental support, and the training environment, which could affect actual intervention completion. This interpretation is consistent with the attrition reported in some of the long-term resistance training studies included in the present meta-analysis, suggesting that training interruptions or reduced adherence may attenuate the potential cumulative benefits of prolonged training. Furthermore, it should be noted that children with special needs (e.g., those with Down syndrome or cerebral palsy) may experience greater functional benefits from resistance training due to their lower baseline motor capacity and functional limitations. In addition, training programs designed for these populations are typically individualized and implemented under professional supervision, which may help ensure intervention quality and safety and thereby enhance training effectiveness. However, the intervention effects may be influenced by multiple interacting factors, including training content, implementation quality, and baseline functional status. Further research is therefore warranted to clarify these potential determinants. Overall, from a clinical perspective, the present study provides preliminary evidence that resistance training may confer functional benefits for postural control in children and adolescents. However, the effects of training duration, developmental stage, and intervention characteristics on training outcomes require further investigation. These findings have potential implications for school-based physical activity interventions and pediatric rehabilitation practice. In contexts where improving participation or enhancing adherence is a priority, short-term, goal-oriented resistance training may serve as a feasible initial intervention strategy. For children with special needs requiring long-term functional rehabilitation, individualized, progressive training programs tailored to baseline functional capacity should be developed to facilitate sustained improvements in motor performance.

This study has several limitations that should be acknowledged. ① Only studies published in English were included, which may have introduced potential language bias and limited the comprehensiveness and generalizability of the available evidence. ② Some included studies had methodological limitations in randomization, allocation concealment, blinding, and outcome assessment. These limitations may have affected the accuracy of the pooled estimates, and the certainty of evidence was downgraded due to concerns regarding the risk of bias. ③ Individual participant data were not available, limiting the ability to adequately account for potential confounding factors, including differences in baseline characteristics and adherence to intervention protocols. ④ Variations in the content and intensity of interventions received by control groups may have contributed to between-study heterogeneity and influenced the pooled effect estimates. ⑤ The limited number of studies available for some analyses reduced the statistical power to assess publication bias and may have affected the precision of the effect estimates. ⑥ Different balance assessment tools were used across studies. Although SMDs were applied to standardize outcomes, differences in measurement properties among instruments cannot be completely excluded. ⑦ The included studies involved participants across different developmental stages, including preschool children, school-aged children, and adolescents. Differences in neuromuscular maturation, baseline postural control ability, motor experience, and adaptation to resistance training across developmental stages may have contributed to clinical and methodological heterogeneity.

## 5 Conclusion

Resistance training may improve both static and dynamic balance performance in children and adolescents. These findings should be interpreted cautiously due to methodological limitations, as well as heterogeneity in intervention characteristics and study populations. Different resistance training modalities may have varying effects on dynamic balance, with core-focused and whole-body resistance training approaches demonstrating relatively greater benefits. Future randomized controlled trials using standardized resistance training protocols and consistent outcome assessment methods are warranted to further elucidate the effects of resistance training on postural control development.

## Data Availability

The raw data supporting the conclusions of this article will be made available by the authors, without undue reservation.
