# Supplementary material for: The effects of resistance training on postural control in children and adolescents: a systematic review and meta-analysis
Source: Front Physiol. 2026 Oct 28;17:1928723. doi: 10.3389/fphys.2026.1928723 (PMC13751337; doi:10.3389/fphys.2026.1928723)
Supplement: Supplementary file 1 [file DataSheet1.docx]

Supplementary Material

**Table 1:Search strategy：**

**1.1 PubMed (n=30)**

#1"Resistance Training"[Mesh]
#2(((((((((((Strength Training[Title/Abstract]) OR (Weight-Lifting Strengthening Program[Title/Abstract])) OR (Weight-Lifting Exercise Program[Title/Abstract])) OR (Weight Lifting Exercise Program[Title/Abstract])) OR (Weight Bearing Strengthening Program[Title/Abstract])) OR (Weight-Bearing Exercise Program[Title/Abstract])) OR (strength training[Title/Abstract])) OR (Weight-Lifting[Title/Abstract])) OR (strengthening program[Title/Abstract])) OR (weight bearing strengthening program[Title/Abstract])) OR (weight bearing exercise program[Title/Abstract])) OR (weight bearing exercise program[Title/Abstract])
#3("Resistance Training"[Mesh]) OR ((((((((((((Strength Training[Title/Abstract]) OR (Weight-Lifting Strengthening Program[Title/Abstract])) OR (Weight-Lifting Exercise Program[Title/Abstract])) OR (Weight Lifting Exercise Program[Title/Abstract])) OR (Weight Bearing Strengthening Program[Title/Abstract])) OR (Weight-Bearing Exercise Program[Title/Abstract])) OR (strength training[Title/Abstract])) OR (Weight-Lifting[Title/Abstract])) OR (strengthening program[Title/Abstract])) OR (weight bearing strengthening program[Title/Abstract])) OR (weight bearing exercise program[Title/Abstract])) OR (weight bearing exercise program[Title/Abstract]))

#4"Child"[Mesh]
#5((((Children[Title/Abstract]) OR (Adolescents[Title/Abstract])) OR (Teenagers[Title/Abstract])) OR (Youth[Title/Abstract])) OR (Pediatric[Title/Abstract])
#6("Child"[Mesh]) OR (((((Children[Title/Abstract]) OR (Adolescents[Title/Abstract])) OR (Teenagers[Title/Abstract])) OR (Youth[Title/Abstract])) OR (Pediatric[Title/Abstract]))
#7"Postural Balance"[Mesh]
#8(postural control[Title/Abstract]) OR (body equilibrium[Title/Abstract])
#9("Postural Balance"[Mesh]) OR ((postural control[Title/Abstract]) OR (body equilibrium[Title/Abstract]))

#10((randomized controlled trial[Publication Type]) OR (randomized[Title/Abstract])) OR (placebo[Title/Abstract])

#11(((("Resistance Training"[Mesh]) OR ((((((((((((Strength Training[Title/Abstract]) OR (Weight-Lifting Strengthening Program[Title/Abstract])) OR (Weight-Lifting Exercise Program[Title/Abstract])) OR (Weight Lifting Exercise Program[Title/Abstract])) OR (Weight Bearing Strengthening Program[Title/Abstract])) OR (Weight-Bearing Exercise Program[Title/Abstract])) OR (strength training[Title/Abstract])) OR (Weight-Lifting[Title/Abstract])) OR (strengthening program[Title/Abstract])) OR (weight bearing strengthening program[Title/Abstract])) OR (weight bearing exercise program[Title/Abstract])) OR (weight bearing exercise program[Title/Abstract]))) AND (("Child"[Mesh]) OR (((((Children[Title/Abstract]) OR (Adolescents[Title/Abstract])) OR (Teenagers[Title/Abstract])) OR (Youth[Title/Abstract])) OR (Pediatric[Title/Abstract])))) AND (("Postural Balance"[Mesh]) OR ((postural control[Title/Abstract]) OR (body equilibrium[Title/Abstract])))) AND (((randomized controlled trial[Publication Type]) OR (randomized[Title/Abstract])) OR (placebo[Title/Abstract]))

**1.2 Embase (n=398)**

#1'resistance training'/exp OR 'resistance training' OR (('resistance'/exp OR resistance) AND ('training'/exp OR training))
#2 strength AND training

#3'weight lifting AND strengthening AND program'

#4'weight lifting' AND strengthening

#5'weight lifting'

#6weight AND bearing AND exercise AND program

#7'child'

#8children

#9'adolescents'

#10'teenagers'

#11'youth'
#12'pediatric'
#13body AND equilibrium
#14'postural balance'/exp OR 'postural balance' OR (postural AND ('balance'/exp OR balance))
#15postural AND control

#16randomized controlled trial
#17rct

#18 #1 OR #2 OR #3 OR #4 OR #5 OR #6
#19 #7 OR #8 OR #9 OR #10 OR #11 OR #12

#11 #13 OR #14 OR #15
#12 #16 OR #17

**1.3 Cochrane library (n=25)**

#1 MeSH descriptor: [Resistance Training] explode all trees

#2 ("Resistance Training":ti,ab,kw OR "Strength Training":ti,ab,kw)

AND

("Children":ti,ab,kw OR "Adolescents":ti,ab,kw OR "Teenagers":ti,ab,kw OR "Youth":ti,ab,kw OR "Pediatric":ti,ab,kw)

AND

("Postural balance":ti,ab,kw OR "body equilibrium":ti,ab,kw OR "Postural control":ti,ab,kw)

AND

("Randomized Controlled Trial":ti,ab,kw OR "RCT":ti,ab,kw)

**1.4 Scopus (n=166)**

TITLE-ABS-KEY(resistance training) OR

TITLE-ABS-KEY(strength training) AND

TITLE-ABS-KEY(children) OR TITLE-ABS-KEY(adolescents) OR TITLE-ABS-KEY(teenagers) OR

TITLE-ABS-KEY(pediatric) OR

TITLE-ABS-KEY(youth) AND

TITLE-ABS-KEY(postural) AND

TITLE-ABS-KEY(balance) OR

TITLE-ABS-KEY(body equilibrium) AND

TITLE-ABS-KEY(randomized) AND

TITLE-ABS-KEY(controlled) AND

TITLE-ABS-KEY(trial) OR

TITLE-ABS-KEY(rct)

**1.5 Web of science (n=34)**

TS=("Resistance Training" OR “Strength Training")

AND TS=("Children" OR "Adolescents" OR "Teenagers" OR "Child" OR "Adolescent" OR "Pediatric" )

AND TS=("Postural Balance" OR "body equilibrium" OR "Postural Control" )

AND TS=("Randomized Controlled Trial" OR "RCT" OR "randomized trial" OR "controlled trial")

**Table 2. Summary of records excluded during title and abstract screening**

| **Screening stage** | **Number**  **excluded** | **Main reason** |
| --- | --- | --- |
| Title screening | 258 | Not related to resistance training, postural balance, or children population. |
| Abstract screening | 83 | Did not meet the predefined eligibility criteria based on population, intervention, comparator, or outcomes. |

**Table 3: Detailed justification for risk of bias assessment using the RoB 2 tool.**

| **Study** | **Domain** | **Judgement** | **Supporting rationable** |
| --- | --- | --- | --- |
| Tuncer 2025 | Deviations from the intended interventions | Some concerns | During the trial, nursing staff and those delivering the interventions were aware of the interventions to which participants had been allocated. |
|  | Measurement of the outcome | High | As the outcome assessors are aware of the interventions received by the study participants, the assessment of outcomes may be influenced by the known interventions. |
| Ozmen 2015 | Randomisation process | High | No specific method for concealing the allocation of randomised groups has been provided. |
|  | Deviations from the intended interventions | Some concerns | During the trial, nursing staff and those delivering the interventions were aware of the interventions to which participants had been allocated. |
|  | Measurement of the outcome | High | As the outcome assessors are aware of the interventions received by the study participants, the assessment of outcomes may be influenced by the known interventions. |
| Granacher 2011 | Randomisation process | High | No specific method for concealing the allocation of randomised groups has been provided. |
|  | Deviations from the intended interventions | Some concerns | During the trial, nursing staff and those delivering the interventions were aware of the interventions to which participants had been allocated. |
|  | Measurement of the outcome | High | As the outcome assessors are aware of the interventions received by the study participants, the assessment of outcomes may be influenced by the known interventions. |
| Elnaggar 2022 | Deviations from the intended interventions | Some concerns | During the trial, nursing staff and those delivering the interventions were aware of the interventions to which participants had been allocated. |
| Larsen 2016 | Deviations from the intended interventions | Some concerns | During the trial, nursing staff and those delivering the interventions were aware of the interventions to which participants had been allocated. |
| Park 2024 | Deviations from the intended interventions | Some concerns | During the trial, nursing staff and those delivering the interventions were aware of the interventions to which participants had been allocated. |
| El-Shamy 2017 | Deviations from the intended interventions | Some concerns | During the trial, nursing staff and those delivering the interventions were aware of the interventions to which participants had been allocated. |
|  | Measurement of the outcome | High | As the outcome assessors are aware of the interventions received by the study participants, the assessment of outcomes may be influenced by the known interventions. |
| Elnaggar 2021 | Deviations from the intended interventions | Some concerns | During the trial, nursing staff and those delivering the interventions were aware of the interventions to which participants had been allocated. |
|  | Measurement of the outcome | High | As the outcome assessors are aware of the interventions received by the study participants, the assessment of outcomes may be influenced by the known interventions. |
| Alsakhawi 2019 | Randomisation process | High | No specific method for concealing the allocation of randomised groups has been provided. |
|  | Deviations from the intended interventions | Some concerns | During the trial, nursing staff and those delivering the interventions were aware of the interventions to which participants had been allocated. |
|  | Measurement of the outcome | High | As the outcome assessors are aware of the interventions received by the study participants, the assessment of outcomes may be influenced by the known interventions. |
| Aly 2016 | Deviations from the intended interventions | Some concerns | During the trial, nursing staff and those delivering the interventions were aware of the interventions to which participants had been allocated. |
|  | Measurement of the outcome | High | As the outcome assessors are aware of the interventions received by the study participants, the assessment of outcomes may be influenced by the known interventions. |
| Hammami 2019 | Randomisation process | High | No specific method for concealing the allocation of randomised groups has been provided. |
|  | Deviations from the intended interventions | Some concerns | During the trial, nursing staff and those delivering the interventions were aware of the interventions to which participants had been allocated. |
|  | Measurement of the outcome | High | As the outcome assessors are aware of the interventions received by the study participants, the assessment of outcomes may be influenced by the known interventions. |

**Table 4：PEDro for resistance training on postural control of teenagers.**

| Included studies | Eligibility and source  **(not included in total score)** | Random allocation | Concealed allocation | Groups similar at baseline | Participants blinding | Therapist blinding | Assessor blinding | outcome measures were obtained from at least 85% of the participants | Intension to treat analysis | Between group difference reported | Point estimate and variability reported | Total | Quality assessment |
| --- | --- | --- | --- | --- | --- | --- | --- | --- | --- | --- | --- | --- | --- |
| Tuncer 2025 | 1 | 1 | 1 | 1 | 0 | 0 | 0 | 1 | 1 | 1 | 1 | 7 | Moderate |
| Kordi  2016 | 1 | 1 | 1 | 1 | 0 | 0 | 0 | 1 | 1 | 1 | 1 | 7 | Moderate |
| Ozmen 2016 | 1 | 1 | 0 | 1 | 0 | 0 | 0 | 1 | 1 | 1 | 1 | 6 | Moderate |
| Granacher 2011 | 1 | 1 | 0 | 1 | 0 | 0 | 0 | 1 | 1 | 1 | 1 | 6 | Moderate |
| Elnaggar 2022 | 1 | 1 | 1 | 1 | 0 | 0 | 1 | 1 | 1 | 1 | 1 | 8 | Moderate |
| Larsen 2016 | 1 | 1 | 1 | 1 | 0 | 0 | 0 | 1 | 1 | 1 | 1 | 7 | Moderate |
| Park  2024 | 1 | 1 | 1 | 1 | 1 | 0 | 1 | 1 | 1 | 1 | 1 | 9 | High |
| EI-Shamy 2017 | 1 | 1 | 1 | 1 | 0 | 0 | 0 | 1 | 1 | 1 | 1 | 7 | Moderate |
| Alsakhawi 2019 | 1 | 1 | 1 | 1 | 0 | 0 | 0 | 1 | 1 | 1 | 1 | 7 | Moderate |
| Aly 2016 | 1 | 1 | 1 | 1 | 0 | 0 | 0 | 1 | 1 | 1 | 1 | 7 | Moderate |
| Elnaggar 2021 | 1 | 1 | 1 | 1 | 0 | 0 | 0 | 1 | 1 | 1 | 1 | 7 | Moderate |
| Hammami 2019 | 1 | 0 | 0 | 1 | 0 | 0 | 0 | 1 | 1 | 1 | 1 | 5 | Low |

**Table 5:**Characteristics of the studies included in the review.

| Included studies | Age | Country | Sample Size | | Interventions | | Intervention frequent | Intervention times |
| --- | --- | --- | --- | --- | --- | --- | --- | --- |
|  |  |  | T | C | T | C |  |  |
| Tuncer 2025 | 4--7 | Turkey | 18 | 18 | RT（Intrinsic and extrinsic muscle strengthening exercises） | intrinsic training | 5 | 8 |
| Kordi 2016 | T:8.01±0.54 | Iran | 15 | 15 | RT | physical education | 2 | 12 |
|  | C:7.70±0.63 |  |  |  |  |  |  |  |
| Ozmen 2016 | 10.8±0.3 | Turkey | 10 | 10 | RT（Core strength exercises） | physical training | 2 | 6 |
| Granacher 2011 | T:16.7±0.6 | Gramany | 14 | 14 | RT | physical education | 2 | 8 |
|  | C:16.8±0.7 |  |  |  |  |  |  |  |
| Elnaggar 2022 | T:13.17±2.10 | Egypt | 18 | 18 | RT（Accommodating variable-resistance exercise） | physical therapy | 3 | 8 |
|  | C:12.72±2.46 |  |  |  |  |  |  |  |
| Larsen 2016 | 9.3±0.3 | United Kingdom | 83 | 116 | RT（Cycle strength training） | small-sided ball game | 3 | 40 |
| Park 2024 | T:14.1±0.7 | Korea | 17 | 17 | RT（strength training） | physical therapy | 3 | 6 |
|  | C:14.4±0.7 |  |  |  |  |  |  |  |
| EI-Shamy 2017 | T:10.26±0.79 | Saudi Arabia | 15 | 16 | RT | physical therapy | 3 | 12 |
|  | C:10.05±0.68 |  |  |  |  |  |  |  |
| Elnaggar 2021 | T:13.5±0.3 | Saudi Arabia | 21 | 21 | RT（stretch-shortening cycle exercises） | standard physical rehabilitation | 2 | 8 |
|  | C:13.3±0.3 |  |  |  |  |  |  |  |
| Alsakhawi 2019 | 4.59±0.53 | Egypt | 15 | 15 | RT（Core stability exercises） | physical training | 3 | 8 |
| Aly 2016 | T:8.11±1.26 | Egypt | 15 | 15 | RT（Core stability exercises） | physical therapy | 3 | 8 |
|  | C:8.34±1.07 |  |  |  |  |  |  |  |
| Hammami 2019 | T:10.43±1.16 | Canada | 21 | 20 | RT（combined upper- and lower-limb plyometric training） | physical training | 2 | 9 |
|  | C:9.86±1.39 |  |  |  |  |  |  |  |

**Table 6** Quality of evidence using the GRADE.

| Outcome | Risk of Bias | Inconsistency | Indirectness | Imprecision | Publication | Certainty of evidence |
| --- | --- | --- | --- | --- | --- | --- |
| Static balance | Serious^a^ | Not serious | Serious^c^ | Serious^d^ | Not downgraded^e^ | Very low⊕◯◯◯ |
| Dynamic balance | Serious^a^ | Serious^b^ | Serious^c^ | Serious^d^ | Not downgraded^e^ | Very low⊕◯◯◯ |
| GRADE Working Group grades of evidence **High certainty** (⊕⊕⊕⊕): We are very confident that the true effect lies close to that of the estimate of the effect. **Moderate certainty** (⊕⊕⊕◯): We are moderately confident in the effect estimate: the true effect is likely to be close to the estimate of the effect,but there is a possibility that it is substantially different. **Low certainty** (⊕⊕◯◯): Our confidence in the effect estimate is limited: the true effect may be substantially different from the estimate of the effect. **Very low certainty** (⊕◯◯◯): We have very little confidence in the effect estimate: the true effect is likely to be substantially different from the estimate of effect.  Static balance was downgraded due to the limited number of studies and the prevalence of small-sample studies. | | | | | | |
|  |  |  |  |  |  |  |
|  |  |  |  |  |  |  |
|  |  |  |  |  |  |  |
|  |  |  |  |  |  |  |
|  |  |  |  |  |  |  |
|  |  |  |  |  |  |  |
|  |  |  |  |  |  |  |
| a. Downgrade one level due to concerns regarding risk of bias in the included studies. | | | | | | |
| b. Downgraded one level due to substantial heterogeneity among studies. | | | | | | |
| c. Downgraded one level due to concerns regarding indirectness of the evidence. | | | | | | |
| d. Downgraded one level due to imprecision caused by small sample sizes and wide confidence intervals. | | | | | | |
| e: Publication bias was not downgraded because a comprehensive search strategy was applied without restrictions based on publication status or publication type. However, the possibility of publication bias cannot be completely excluded due to the limited number of included studies. | | | | | | |

**Figure 1: Leave-one-out sensitivity analysis for the effect of resistance training on dynamic balance outcomes.**

**A:** Influence analysis showing the pooled effect estimates and 95% confidence intervals after sequentially excluding each individual study. **B:** Forest plot of leave-one-out sensitivity analysis. Each line represents the pooled effect estimate obtained after removing one study at a time, indicating that no single study substantially altered the overall effect estimate.

**Figure 2: Sensitivity analysis excluding studies with lower methodological quality**

**Figure 3: Population-based sensitivity analyses**

**A:** Pooled effect estimate after retaining studies involving clinical populations only (6 studies; SMD=0.89, 95%CI[0.28,1.50]).

**B:** Pooled effect estimate after excluding clinical populations and retaining studies involving healthy children/adolescents or athletes only (6 studies; SMD=0.30, 95%CI[-0.01,0.61]).

**Figure 4: Comparator-based sensitivity analyses**

**A:** Effect estimate from studies comparing resistance training with conventional physical therapy.

**B:** Effect estimate from studies comparing resistance training with usual training or physical education classes.

**Figure 5: Sensitivity analysis excluding preschool-aged participants for dynamic balance.**
